# Focused ultrasound and immunotherapy

**DOI:** 10.1186/2050-5736-3-S1-O42

**Published:** 2015-06-30

**Authors:** Mark Hurwitz

**Affiliations:** 1 Jefferson University Hospitals, Philadelphia, Pennsylvania, United States

## Background/introduction

Thermal therapy has tremendous potential to augment the benefits of immunotherapy. The ability of heat to stimulate both general and tumor specific responses, in part through heat shock protein mediated mechanisms has been known for over two decades. Significant challenges persist however in translating these effects into predictable and meaningful clinical responses. More recently the association of radiation with immune stimulation and in some cases specific anti-tumor immune response has been defined.

## Methods

Coupled with the introduction of new immunotherapuetics including check point inhibitors which have demonstrated clinical survival benefit, the combination of heat, radiation, and immunotherapy to combat cancer is a timely area for investigation. The potential of focused ultrasound to augment immunotherapy including targeted heat and drug delivery will be discussed including the opportunity to enhance immune effects arising from the heated but non-ablated rim present with tumor ablation.

